# Social COmmunication Program supported by E-health (SCOPE) for infants and toddlers at elevated likelihood of autism spectrum disorder: study design of a cluster randomized controlled trial

**DOI:** 10.1186/s12888-022-04351-x

**Published:** 2022-12-08

**Authors:** Michelle I. J. Snijder, Claudine Dietz, Mieke van Andel, Emilie L. M. Ruiter, Jan K. Buitelaar, Iris J. Oosterling

**Affiliations:** 1 Karakter Child and Adolescent Psychiatry University Centre, Nijmegen, the Netherlands; 2 Department of Cognitive Neuroscience, Donders Institute for Brain, Cognition and Behaviour, Radboudumc, Nijmegen, The Netherlands; 3 GGD Gelderland-Zuid, Nijmegen, The Netherlands

**Keywords:** Autism spectrum disorder, Early intervention, High risk, Parent mediated intervention, BEAR

## Abstract

**Background:**

Although the importance of early detection and early intervention of autism spectrum disorders (ASD) is widely recognized, multiple barriers exist in accessing early intervention services. As an alternative to these barriers, the SCOPE project presents a new, easy accessible and blended intervention called BEAR (Blended E-health for children at eArly Risk). This paper describes this BEAR intervention and study design of an ongoing two arm cluster randomized controlled trial (RCT).

**Methods:**

BEAR (Blended E-health for children at eArly Risk) is a blended e-health intervention, based on evidence-based naturalistic developmental behavioral interventions (NDBI’s) and can be offered to parents and infants/toddlers at high likelihood for ASD. During the ongoing RCT, *N* = 88 high risk infants and toddlers will be cluster randomized over the BEAR intervention and care-as-usual (CAU) conditions. The finalized version of the intervention protocol and study design are presented in this paper. The primary outcome measure is *joint engagement* measured by the Joint Engagement Rating Inventory (JERI) during videotaped parent–child interaction. Secondary outcome measures include severity of ASD symptoms, global level of adaptive functioning, parental well-being, parental skills and satisfaction with healthcare. Also, costs will be estimated from society's perspective. Assessments take place at the start of the study (T1), after eight weeks (T2) and after six months (T3) and include behavioral home observations and parental questionnaires.

**Discussion:**

The SCOPE project aims to contribute to improved early identification and timely start of suitable interventions for infants and toddlers at elevated likelihood for ASD. This ongoing RCT will offer insight in the feasibility, short-term and six months effects of the innovative BEAR intervention. It is estimated that inclusion for the trial (*N* = 88) is completed in spring 2023.

**Trial registration:**

Dutch Trial Register, NTR7695. Registered at December 17^th^, 2018, www.trialregister.nl.

**Supplementary Information:**

The online version contains supplementary material available at 10.1186/s12888-022-04351-x.

## Background

The importance of early detection and intervention of autism spectrum disorder (ASD) is widely recognized [1]. Since greater plasticity of the brain during preschool years facilitate learning opportunities, early interventions starting at this crucial period have the best chance of altering neural connectivity [2]. Early intervention during the first three years of life is expected to decrease core autism symptoms [3], enhance social communication between parent and child [4] and improve cognitive and adaptive behavior [5]. Unfortunately, varying time gaps of 1.5 to 3.5 years exist between first raised concerns and an ASD diagnosis [6–8]. So, despite an increased awareness of the importance of early detection and intervention, infants and toddlers at high likelihood for ASD generally receive access to appropriate intervention later than preferred.

Difficulties in early identification and access to early interventions are multifaceted, with barriers related to child, parent, professional and organizational levels [9, 10]. Focusing on *child characteristics*, children with milder symptoms of ASD and (above) average IQ are often not recognized at an early age[11], whereas children with more severe ASD and cognitive impairments are identified sooner [9]. Also, girls and children from ethnic minorities are at risk of late identification [12, 13]. From *parents perspective*, it is sometimes difficult for a parent to acknowledge and accept that their child might develop differently, and therefore parents might be reticent to be referred to specialized mental healthcare when their child is still very young [14]. On the *professional level* (especially for preventive care professionals), limited knowledge about ASD symptoms in infant- and toddlerhood and limited use of screening instruments are main components in late identification, as well as unfamiliarity with the opportunities and advantages of early intervention [6, 15, 16]. Lastly, on *organizational level*, long waiting lists and limited service capacity make it difficult to access early interventions [6, 16, 17]. The lack of accessible early interventions raises the ethical question of why healthcare professional should screen for ASD, if there are no suitable referral options. Furthermore, insufficient compensation (i.e. time and money constraints) for healthcare professionals lead to the absence of investment in additional training (i.e. therefore the lack of knowledge regarding early signs of ASD) and adherence to screening guidelines [16, 18]. So, in order to improve early identification and access to early interventions, integrated improvement strategies targeting both child, parent, professional and organizational levels are highly required.

The SCOPE (Social COmmunication Program supported by E-health) project aims to improve early detection and access to intervention by introducing three components that are developed to overcome aforementioned barriers and will be discussed below (see also Snijder et al., 2021b). First, an informative online platform for parents and professionals was developed (www.autismejongekind.nl). This platform offers easy and accessible information about the early indicators of ASD to parents and professionals and at the same time spreads awareness about the importance of early detection and intervention. Second, preventive care professionals in the target region are trained in recognizing the early signs of ASD. Previous research shows this as an effective way of improving early detection, if continuously invested in [11, 18, 19]. In the Netherlands, preventive care professionals at well-baby clinics are the first healthcare providers to have systematic contact with families, mainly for routine health checks and vaccinations and therefore play a pivotal role in the early detection process of ASD (i.e. signaling, screening and referring). Almost all children aged 0–4 and their parents visit the well-baby clinics (94%; CBS, 2014). Therefore, training in several aspects of early detection of ASD focusses primarily on these preventive care professionals, but also on other important professionals such as general practitioners and pedagogical staff at daycare centers. The third component of SCOPE contains a relatively short and acceptable home-based early intervention (BEAR: Blended E-health for children at eArly Risk) offered to parents with symptomatic high risk infants and toddlers. There might be ASD related concerns regarding the child’s development, but not necessarily a confirmed ASD diagnosis. The BEAR intervention is an preemptive intervention offered by a first line healthcare professional, supervised by a specialized mental healthcare professional (and working within a more specialized setting). A recent systematic review by Hampton and Rodriguez (2021) on preemptive interventions suggest that parent-mediated interventions are associated with better parental use of strategies and although results do not translate one-on-one into short-term developmental outcomes of the child*,* there is a proposition that successful parent implementation facilitate later social communication of the child.

The global focus of the BEAR intervention is helping parents to understand their child’s behavior, promoting sensitivity in parents to their child’s needs and, through that, to motivate the child to socially engage (e.g., improving joint engagement, enhancing initiatives in communication). Theoretically BEAR builds on two well-studied principles: 1) high synchrony between parent and child is assumed to be related to decrease in autism symptoms and 2) improved joint attention and joint engagement skills are related to better communicative abilities [20–22]. Like many early interventions (such as Pivotal Response Treatment, Floorplay and JASPER-training) BEAR intervention techniques are based on evidence-based naturalistic developmental behavioral intervention principles (NDBI’s; [23]). BEAR is meant for children for whom referral for a diagnostic trajectory might not be applicable yet (because of unclear indicators or mild signals), or when serious concerns regarding development exist but parents are not yet ready for referral to a more specialized center for infant psychiatry (see Snijder et al., 2021b). The innovative value of BEAR is not so much in the content or theoretical framework, but rather in the combination of an early start of intervention (pre-diagnosis), easy accessibility (no waiting list and at home), and strong collaboration between first line (executer) and specialized mental healthcare (supervisor). Without the need of an ASD diagnosis, BEAR is presumably more acceptable for those parents who do not yet have great concerns or an explicit question for help, and is at the same time an appropriate form of healthcare for the children who show unclear risk signals. Additionally, for some children this intervention will be the influx to more intensive and specialized healthcare. Considered this way, BEAR might function as a triage agent, whereas for other children BEAR will function as a way of (secondary) prevention.

In the SCOPE project, a cluster randomized controlled trial (RCT) (two armed, 44:44 ratio) will be employed to study the immediate short time effects, effects after six months and cost-effectiveness of the BEAR intervention in a highly indigent target population. The primary objective is to improve joint engagement in the parent–child interaction. Secondary objectives are improving social-communicative development of the child at elevated likelihood of ASD, improving parental skills and well-being and decreasing the gap between first concerns and start of adequate intervention. Before starting a larger cluster RCT, an important first step was to pilot test the intervention in a small sample. Based on insights gained from the pilot sample, potential research problems were identified and solved, in order to fully maximize the potential of a successful cluster RCT. The aims of the current paper are to present (1) the BEAR intervention protocol and (2) the study design of the cluster RCT.

## Methods

### BEAR intervention

BEAR (Blended E-health for children at eArly Risk) is a short and easily accessible, parent adopted and blended e-health intervention. It can be offered to parents and children aged between 12–30 months when first concerns of ASD have been expressed. The intervention is to be delivered by a trained professional working in preventive care, preferably under supervision of a professional working in specialized mental healthcare and considered to be an ASD expert, in order to obtain the best of both worlds (easy access through preventive care and expert knowledge through specialized care) and promote collaboration between different healthcare settings. The BEAR intervention consists of seven home visits and five additional e-learning sessions for parents. The first session is a general introduction module, containing psycho-education for parents about child development on the areas of play, social communication, flexible behavior and sensory interest. Next, BEAR offers five possible intervention modules aimed aforementioned areas, partially based on the DIR/Floortime model [24]. Modules include (1) improving attention to the (play) environment, (2) becoming interested in social contact, (3) increasing social contact and communication, (4) improving social communication and (5) increasing flexible behavior. At its core, BEAR is about following the child's interests, matching the child's developmental level and pace, and ensuring fun in the interaction as the basis for all further learning. A paper version of BEAR is available for parents who prefer it. An outline of the BEAR intervention’s content and planning is provided in Table 1.

**Table 1:** Outline of the content of the BEAR intervention

Home visit	Content	Attendees	Duration
1	A twelve minute play interaction between parent and child will be videotaped, enabling professionals to generally estimate the social and communicative competencies of the child. Together with parents, three specific modules are chosen	Child, parent, BEAR professional ^a^	90 min
2–6	Parents complete e-learning modules accompanied by weekly home visits by the BEAR professional. The e-learning provides a theoretical introduction to the themes that will be discussed, are tailor made to the needs of parent and child and are implemented into practice during the home-visits	Child, parent and BEAR professional	E-learning modules take about 45 min each Home visits take about 60 min
7	The last session is a summary and evaluation of the learning process of child and parents. If serious concerns continue to exist, clinical assessment, diagnostic referral and/or further treatment can be advised. Specialized knowledge from the BEAR supervisor will enable to come up with an appropriate advise	Child, parent, BEAR professional	90 min

^a^The ASD expert supervises remotely throughout the entire process

### Cluster randomized controlled trial

#### Study design and randomization

The effectiveness of the BEAR intervention will be studied in a two-armed cluster RCT (ratio 44:44). Well-baby clinic locations in the Nijmegen area in the Netherlands will be randomly assigned to either the BEAR or Care-as-usual (CAU) condition. Before randomization, the well-baby clinics will first be matched based on two characteristics (amount of children visiting that well-baby clinic and ethnicity/social-economic background of the specific area) to ensure an equal distribution in the two groups. By coin flipping, the well-baby clinics are at random assigned to either the BEAR condition or CAU. Consequently, the study is open-labeled; both participants (children and parents) and the therapist know to which condition the participant is assigned through cluster randomization. However, for the primary outcome measure, our assessors rating parent–child interaction are unaware of group allocation (see Table 2).

**Table 2:** Outcome measures from baseline to follow-up

Measurement	Time^a^	Instrument	Informant	Blind to group status?
**Descriptives**				
Demographics	T1	Single questions	Parent(s)	
IQ	T1	Bayley-III	Clinician	
Problem behavior	T1	BITSEA	Parent(s)	
Parental ASD symptoms	T1	SOV	Parent(s)	
**Primary outcome**				
Joint engagement	T1, T2, T3	JERI	Research assistant (RA) ^b^	Yes
**Secondary outcome**				
Change in ASD symptoms	T1, T2, T3	BOSCC (parent)	RA ^b^	Yes
	T1, T3	BOSCC (clinician)	RA^c^	Yes
	T1, T3	ADOS-2	Clinician	Yes
Expressive language	T1, T2, T3	JERI	RA ^b^	Yes
	T1, T3	N-CDI	Parent(s)	
Adaptive functioning	T1, T3	Vineland Screener	Parent(s)	
Parental well-being	T1, T2, T3	WEMWBS	Parent(s)	
	T1, T2, T3	PSQ	Parent(s)	
Parental skills	T1,T2, T3	JERI	RA ^b^	Yes
`Satisfaction	T2	BESTE-O	Parent(s)	
	T2	BESTE-H	BEAR professional	
	T3	Satisfaction survey	Parent(s)	
Cost measurements	T1, T2, T3	Tic-P	Parent(s)	

*Bayley-III* Bayley Scales of Infant and Toddler Development, *BITSEA* Brief Infant–Toddler Social & Emotional Assessment-Revised, *SOV* Dutch version of the Autism Quotient (AQ), *JERI* Joint Engagement Rating Inventory, *BOSCC* Brief Observation of Social Communication Change, *ADOS-2* Autism Diagnostic Observation Schedule, *N-CDI* Dutch version of the MacArthur Communicative Development Inventory, *Vineland Screener* adapted version of the Vineland Adaptive Behaviour Scales, *WEMWBS* Warwick-Edinburgh Mental Well-being Scale, *PSQ* Parenting Stress Questionnaire (PSQ, in Dutch Opvoedingsbelastingsvragenlijst)*, BESTE* the Rating Scale Satisfaction and Effect questionnaire, *Tic-P* Trimbos and iMTA questionnaire on Costs associated with Psychiatric illness

^a^T1 is baseline; T2 is endpoint, eight weeks after baseline; T3 is follow-up, 24 weeks after endpoint

^b^Both the JERI and the BOSCC(parent) are assessed by the BEAR professional as part of the intervention (only at T1 and T2). At T3, both measurements are assessed by a researcher of the SCOPE project. All data will be coded by research assistants who are unaware of treatment allocation

^c^The BOSCC(clinician) is assessed by a professional examiner. Data will be coded by research assistants who are unaware of treatment allocation

#### Participants

Children at high likelihood of ASD and their families who meet inclusion criteria are recruited at participating well-baby clinics in the allocated cluster region surrounding Nijmegen, the Netherlands. If families outside of the region are interested in participating in the study, participants will be individually randomised in one of the two groups. Children and their parents are eligible to participate if the following criteria are met: a) a screen positive result (≥ 3) on the Communication and Social development Signals (CoSoS, formerly known as ESAT, [25]) list, or with a screen negative result (< 3) although with serious professional and/or parental concern regarding social-communicative development; b) age between 12–30 months; and c) at least one of the parents is able to understand and speak Dutch. Exclusion criteria are family issues that limit the likelihood to engage in an home based intervention, significant chronic illness of the child, severe parental psychopathology (such as depression, psychosis, substance use disorder), a severe intellectual disability of both child and parents, severe vision and hearing impairments and/or severe motor impairments.

#### Procedures

The study consists of two phases with several steps, as described below.

##### Pre-study phase (T0)

- Step 1: Training of professionals
  All preventive care physicians, nurses and other professionals working at the well-baby clinics in the target area first completed an e-learning in recognizing the early symptoms of ASD in infants and toddlers and were trained in administering the CoSoS in order to screen at risk children. Next, preventive care physicians participated in a live online educational program. This program raises physicians level of specific ASD knowledge, as well as their self-confidence in screening for ASD [26]. For their participation, both physicians and nurses were awarded with CME (Continuing Medical Education) points. During training, preventive care physicians and nurses were informed in which study condition their well-baby clinics was assigned to. They received strict instructions from the research team regarding recruitment.
- Step 2: Screening and inclusion
  As part of regular healthcare and screening procedures, the Van Wiechenscheme is conducted at all well-baby clinics to monitor developmental milestones for children from birth to 4 years of age [27]. The surveillance tool holds eight signals, considered to be first behavioral red flags of ASD in infants and toddlers [25]. When one or more behavioral red flags are identified during general surveillance, preventive care physicians and nurses will administer the CoSoS, as recommended in Dutch national screening guidelines [28] and as taught in the training phase. Potential participants that meet inclusion criteria will be orally informed about the study at the well-baby clinics by their healthcare professional. Potential participants will receive a detailed information letter with the aim, content and time investment of the study. Contact information of the research team is provided, so parents can contact them if they have any questions. When parents decide to participate in the study with their child, they are asked to sign an informed consent form and return the form to the research team.

##### Study phase

- Step 1: Baseline assessments (T1)
- After informed consent, parents will be asked to complete the baseline questionnaires send to them online (see Table 2). In addition, a home visit will be planned in order to film the parent–child interaction, perform semi-structured observations of the child, and testing of cognitive abilities (see Table 2).
- Step 2: Intervention
- Within two weeks after baseline assessments, children and parents allocated to the BEAR intervention group will start with the intervention. The care-as-usual group receives either no intervention or care that is normally organized in specific cases (see Interventions section below).
- Step 3: End point assessments (T2)
- About eight to ten weeks after baseline, endpoint measures will be conducted. Parents will be asked to complete questionnaires online (see Table 2). Additionally, parents in the BEAR condition will be asked to complete an extra questionnaire to receive feedback on several aspects of the new parent training. Semi-structured observations of the child and parent–child interaction will be repeated.
- Step 4: Follow-up assessments (T3)
- Follow-up measures will take place 6 months after endpoint (see Table 2). For the last time, parents will be requested to complete online questionnaires and semi-structured observations of the child and parent–child interaction will be conducted.

#### Interventions

##### BEAR condition

A comprehensive description of the BEAR intervention offered to parents and children is described above and can be found in Table 1.

##### Care-as-usual (CAU)

The control condition (CAU) includes regular care trajectories for young children with (signs of) ASD. These trajectories are highly variable and depend on the severity of symptoms, parental wishes and preferences and available services in the specific region. Examples are speech therapy or physiotherapy, referral to an audiology center, but also referral to a specialized daycare center or a referral for clinical assessment and treatment offered by a specialized healthcare center for infant psychiatry. Also it is not uncommon that, after signs of ASD have been identified, a wait-and-see approach is chosen by parents and/or professionals. In that case, CAU could also mean no treatment is offered to children and parents in the control condition.

#### Outcomes

An overview of the study parameters and how they will be assessed can be found in Table 2.

##### Baseline measures

During baseline (T1), the following data will be collected.

**Demographics**

Demographic characteristics (i.e. information about ethnics, parental and/or sibling psychiatry and education levels) are recorded via single questions that parents complete online.

**Intelligence quotient (IQ)**

For the cognitive developmental level of the child, the cognition scale of the Bayley Scales of Infant and Toddler Development (Bayley-III; [29]) will be conducted. Index scores will be reported. Test–retest and inter-examiner reliability show good results. Dutch norms are available and the instrument is deemed as valid.

**Problem behavior**

Problem behavior is measured by the Brief Infant–Toddler Social & Emotional Assessment-Revised (BITSEA; [30]), a short questionnaire sensitive to social-emotional and behavioral problems, autism spectrum disorders, and delays in social-emotional competence in early childhood. It consists of 42 items, rated across a 3-point Likert scale. A total score will be calculated. The BITSEA has excellent test–retest reliability and good inter-rater agreement [30].

**Parental traits of autism**

Parental traits of autism are measured by the self-report questionnaire Social Interaction in Adults (in Dutch: Sociale Omgang bij Volwassenen [SOV]), a Dutch questionnaire developed by Bralten et al. [31] completed by both parents. This questionnaire is derived from items of the Autism Spectrum Quotient and of the DSM-IV section on ASD. The self-report questionnaire consists of 18 items (total scores will be calculated) and has satisfactory internal consistency (Cronbach’s α = 0.70).

#### Primary outcome

##### Joint engagement in the parent–child interaction

A semi-structured 12 min videotaped interaction between parent and child will be collected for each dyad at T1, T2 and T3. A standardized set of toys in two boxes (A and B) will be presented. Parents will be asked to engage in free play with box A for four minutes, followed by blowing bubbles for two minutes. Next, parents are asked to repeat the aforementioned, but now with the box B. The videotapes will be coded by observers blind to the group status and scored for the time and quality spend in different engagement states using Joint Engagement Rating Inventory (JERI; [32]). Recordings will be subsequently coded for four engagement states (total joint engagement, supported joint engagement, coordinated joint engagement and symbol-infused joint engagement). The four items are each defined with 7 points providing information about both quantity and quality of engagement states. The low anchor [1] indicates that there are no episodes of joint engagement during the interaction, whereas the high anchor [7] indicates that the child almost always spends time in the joint engagement state during interaction. The midpoint of 4 characterizes a child who is in joint engagement for approximately half of the scene in several brief or a few relatively sustained episodes. Inter-rater reliability between students/research assistants is deemed reliable when there is a minimum of 80% agreement between observers, based on 15% percent of the tapes. The first author (MS) was trained by one of the developers of the JERI until high accuracy was obtained on all four joint engagement states. For the RCT, students and research assistants will be trained by MS in coding video records with use of the JERI.

#### Secondary outcomes

##### Social-communicative development

Change in social-communicative development is measured by the measured by the Brief Observation of Social Communication Change (BOSCC; [33]). At T1, T2 and T3, BOSCC will be rated based on the same twelve minutes videotaped parent–child dyadic interaction as the joint engagement measure (BOSCCparent). At T1 and T3, an additional BOSCC will be conducted by a skilled examiner. Here, the child is interacting with a professional examiner who has not been part of the intervention (BOSCCprofessional). The BOSCC consists of 15 coding items associated with key features of ASD, such as making eye contact, unusual sensory interests and the frequency and function of social overtures. The difference in total score between measurement moments is indicative for change in behaviour in the social-communicative domain. The BOSCC is a promising outcome measure and is derived from the Autism Diagnostic Observation Schedule-2 (ADOS-2; [34]) – the “golden standard” measure that has long been used as outcome measure in early intervention studies. Research shows that this new instrument has satisfactory inter-and intra-rater reliability [18, 33]. Next, the ADOS-2 (toddler, module 1 and/or module 2) will be administered at T1 and T3. The ADOS-2 is a semi-structured play observation, where the clinician elicits social, communicative, stereotyped and play behaviour to observe symptoms of ASD. Observations of the clinician are categorized and a score is assigned for each domain of ASD symptoms. Although the ADOS-2 has thus long been considered the “golden standard” in intervention studies, the instrument has its limitations. For example, the ADOS-2 can identify changes in ASD symptoms over a couple of years [3] but due to the narrow range of scores used for each item, the ability to detect subtle changes in behaviour over a shorter time frame might be limited. Also, the ADOS has not been developed to quantify different degrees of autism severity, rather it has been developed to allow for a diagnostic algorithm (yes/no autism). The BOSCC might be more sensitive in detecting subtle changes and better suitable to quantify autism severity. However, since the BOSCC is a relatively new instrument, both instruments will be administered in this study (also enabling future in-depth instrument comparisons).

**Language**

The child’s expressive level of language will be measured by the Dutch adaptation of the MacArthur Communicative Development Inventory: Toddler (N-CDI; Fenson et al., [35, 36]), a parent report, at T1 and T3. Raw scores will be calculated as to indicate both language production and language comprehension. Also, level of expressive language will be measured by using the JERI. Based on the same twelve minute dyadic videotape child’s expressive language level and use will be rated from 1 (no expressive language) to 7 (fluent and frequent use of sentences) at T1, T2 and T3.

**Global level of adaptive functioning**

For estimating global level of adaptive functioning, the Vineland Screener will be conducted at T1 and T3. The Vineland Screener is an adapted Dutch version of the Vineland Adaptive Behaviour Scales (VABS: Sparrow et al., 2005 [37, 38]), consisting of 90 items, to be filled out by one parents/primary caregiver. It consists of the following scales: Communication domain, Daily Skills domain, Socialization domain and Motor Skills domain. Each of the items in the previous mentioned domains contains a statement of child adaptive behavior. Subscales will be calculated as to identify change in global level of adaptive functioning at starting point (T1) and at follow up (T3). Parents themselves rate whether the child mostly (2), sometimes/partly (1) or never (0) performs the behavior or action independently.

**Parental well-being**

Parental well-being is measured by the Warwick-Edinburgh Mental Well-being Scale (WEMWBS; [39]) and Parenting Stress Questionnaire (PSQ, in Dutch Opvoedingsbelastingsvragenlijst, OBVL; [40]). The WEMWBS is a reliable instrument that measures mental well-being. It consists of 14 items, measured on a 5-point Likert type scale and will be completed by both parents/primary caregivers. Additionally, the PSQ is used to determine parental experiences with their child, how they interact with their child and how parents feel about their own health. The PSQ is a well validated and reliable questionnaire that consists of 34 items on a 4-point scale ranging from ‘not true’ to ‘very true’. For both instruments, total scores will be calculated during all measurement points.

**Parental skills**

Parental intervention skills, or parental fidelity is defined as “to execute parent-implemented techniques accurately and consistently” [41]. By measuring parental skills, investigators document that parents can indeed perform the intervention techniques as they were intended to be used. These skills will be rated by the parent scales of the JERI and consists of four items covering caregivers’ scaffolding, symbol highlighting, following in on child’s focus and caregivers’ affect. The scaffolding item assesses how well the parent supports the child’s activities and provides learning opportunities. Symbol highlighting focuses on how often the caregiver directs the child’s attention to symbols (language and/or symbolic gestures and acts). Following in on child’s focus captures if the parent is following the child’s interests and maintain focus with the child. Finally, caregivers’ affect measures the parent’s affect and how it influences the parent–child interaction [32]. Items fit the techniques taught by the BEAR intervention. Just as with the joint engagement items, parent scale items will be rated from a scale of 1 to 7. Parental skills will be rated based on the same videotaped dyadic interaction as the joint engagement measure, as collected at baseline, end of treatment and follow-up. Also, in order to capture the flow of interaction between parent and child, an interaction item (fluency and connectedness) will be scored.

**Parental satisfaction**

Parental satisfaction with care in general is measured at follow-up through a survey created by our group. The full survey can be found in the supplementary materials (Additional file 1: Appendix A). Topic items included initial concerns, searching for help, receiving a diagnosis, child and parent treatment and overall satisfaction with the healthcare process. To measure parental satisfaction specifically for the BEAR intervention specifically, the Rating Scale Satisfaction and Effect questionnaire (in Dutch: Beoordelingsschaal Tevredenheid en Effect [BESTE]; [42]) will be conducted. The BESTE consists of two versions: one for parents and one for healthcare practitioners, and both versions will be administered. Validity and reliability have been established [42]. The BESTE will only be conducted end of treatment, and in the BEAR condition only. Descriptive statistics will be used to present percentages regarding satisfaction and effect, as mentioned by both parents and practitioners.

**Assessment of healthcare sources**

Direct and indirect costs as a consequence of the child’s psychiatric condition, i.e. the medical costs and productivity losses in parents are measured using the ‘Trimbos and iMTA questionnaire on Costs associated with Psychiatric illness’ (Tic-P questionnaire; [43]). Validity and reliability have been established [43]. For every participant, the duration and type of each contact as well as the type of health care worker with whom the contact was in the last 3 months will be registered as to monitor use of healthcare sources in both groups as well as to calculate the cost-effectiveness of arms. Productivity losses of parents associated with their child’s health problem or its treatment will be registered as well.

#### Sample size and power

The justification of sample size is calculated based on the primary hypothesis that the BEAR intervention will improve the child’s total joint engagement (measured on a 7-point Likert scale) at the end of treatment. In a comparable study by Kasari et al. [44] with joint engagement as the primary outcome as measured in total seconds spent in joint engagement, a Cohen’s d effect size of 0.21 was found in difference in joint engagement at endpoint. We assume our effect size to be slightly lower than in the study of Kasari et al., due to a shorter duration of the BEAR intervention and the use of an ordinal variable instead of an continuous one. Based on the variance of the treatment effect, a power analysis on an alpha level of 0.05 (two sided) lead to an estimated sample size of 40 participants per group, with a power of 0.88. To allow for 10% drop out 44 participants per group (total *N* = 88) will be recruited.

#### Data collection and management

Data collection follows the European General Data Protection Regulation. After inclusion, participants will receive an unique code number in order to anonymize all data. Non-anonymous data, for example informed consent forms, are locked in a closed cabinet and saved in protected folders, only accessible by appointed members of the research team. In order to collect and store data from the questionnaires, Castor Electronic Data Capture (Castor EDC, 2021) and QuestManager (version 5.6) software are used. Video-data will be anonymously saved on an external hard disk, which is password protected and saved in a locker.

Parents of participants are asked to complete a number of online questionnaires (see Table 2) through the QuestManager software. Parents are only able to continue to a next questionnaire if all items are answered. To prevent missing data, a researcher will call parents as a reminder and offer support if parents encountered any problems, if they fail to complete the measurements on time. Parents may withdraw from the study at any time for any reason. If this happens, researchers will try to make a final appointment with parents in order to collect the primary outcome.

#### Statistical analysis

Data will be analyzed and reported in accordance with CONSORT guidelines. Baseline demographics (IQ, gender, age) and clinical characteristics (severity of symptoms as measured by the CoSoS) of the BEAR and CAU groups will be compared by chi-squared tests to check whether cluster randomization has led to two even groups. For the cluster RCT, all primary analyses will be intention to treat (ITT) using (generalized) linear mixed effects model for repeated measures with a random effect for cluster. Correlation of measurements (T1, T2, T3) within subject will primarily be modelled by a random effect of subject nested within clusters unless this results in insufficient fit, in which case other covariance structures will be investigated. Fixed effect in the model for baseline covariates, time and interaction with treatment condition will be included. For cost effectiveness analysis, all cost data will be accumulated. Cost differences between the two conditions will be compared. Incremental cost-effectiveness ratios will be calculated by dividing the difference in total costs by the difference in the WEMWBS.

Data collection started just before the COVID-19 pandemic. During a lockdown period where home visits were not possible because of COVID-19 restrictions, small changes were made in collecting the data and providing the BEAR intervention. Due to this, a sensitivity analysis will be undertaken, excluding participants included during the first lockdown.

#### Data monitoring

Adverse events (AE’s) reported by the parent/primary caregiver of the participant or observed by the research team or BEAR professional will be recorded. If a serious AE occurs, the researchers will report to the ethics committee within fifteen days of first knowledge of the AE. All AEs will be followed until they have abated, or until a stable situation has been reached. As adverse events resulting from study participation are very unlikely, review or advice of a Data Safety Monitoring Board or a safety committee is not required for the current study. In accordance with the European General Data Protection Regulation, an independent data protection officer is appointed, and monitors the privacy of the participants. The research team (MS and IO) are responsible for processing the data.

## Discussion

The goal of the current study was to describe an innovative early intervention called BEAR and present the finalized study design and methods of the SCOPE study after pilot testing for feasibility. Via a cluster randomized controlled trial with two arms, SCOPE aims to compare the short term and relatively long term effects of BEAR. It is hypothesized that BEAR will lead to improved joint engagement in the parent–child interaction, decreased ASD symptoms, improved parental skills, improved satisfaction of parents with healthcare, a sooner start between first concerns and start of intervention and cost efficiency, both on short and long term (at six months). It is estimated that inclusion for the trial (*N* = 88) is completed in spring 2023 and that follow-up data are completed by the end of 2023.

Please note that the observations from the JERI and BOSCC(parent) are based on the parent–child interaction and that the parents are not an experimental fixed factor since they have been part of the intervention. However, this is not only a limitation but provides also opportunities. For it would allow to disentangle the respective contributions of the parent and the child to any change in the parent–child interaction and the child’s social-communicative behaviour. Furthermore, in addition to the JERI and BOSCC(parent) we will observe the child’s behaviour in the ADOS procedure and a separate BOSCC(clinician) procedure. Here, the child is interacting with a professional examiner who has not been part of the intervention. The analysis of the ADOS and BOSCC(clinician) data is an important back-up to the analysis of the JERI and BOSCC(parent) data by creating the possibility to compare the child’s behaviour while interacting with the parent and with another adult. Furthermore, for our experimental group, there might be bias at our T1 and T2 video measurements, due to the interventionist being the assessor of the JERI and BOSCC. Since data are coded by skilled observers who are not part of the intervention and are unaware of treatment allocation, we hope to minimize this possible bias. This will further be discussed within our finalized paper reporting results of the SCOPE study.

So far, several studies examined the importance and effectiveness of early identification and early intervention programs in high-risk groups. However, still several barriers exist as why very young children at high likelihood of ASD receive adequate care later then preferred. The ongoing SCOPE study in which the BEAR intervention plays a pivotal role can help to overcome these barriers. It is specifically designed as a non-stigmatizing answer to difficulties in those children that are at risk at being identified too late due to a less specific phenotype. Also, when difficulties in parent-professional conversations exist regarding developmental concerns, BEAR might be a solution in offering adequate early care.

## Supplementary Information

**Additional file 1: Appendix A.** Informed Consent.

## Data Availability

The final dataset of the randomized controlled trial will be available (anonymized) for other researchers at the end of the study.

## Abbreviations

ADOS-2: Autism Diagnostic Observation Schedule
AE: Adverse Event
ASD: Autism spectrum disorder
Bayley-III: Bayley Scales of Infant and Toddler Development
BEAR: Blended E-health for children at eArly Risk
BESTE: The Rating Scale Satisfaction and Effect questionnaire
BITSEA: Brief Infant–Toddler Social & Emotional Assessment-Revised
BOSCC: Brief Observation of Social Communication Change
CoSoS: Communication and Social development Signals
JERI: Joint Engagement Rating Inventory
N-CDI: Dutch version of the MacArthur Communicative Development Inventory
PSQ: Parenting Stress Questionnaire (PSQ, in Dutch Opvoedingsbelastingsvragenlijst)
RCT: Randomized controlled trial
SCOPE: Social COmmunication Program supported by E-health
SOV: Dutch version of the Autism Quotient
Vineland Screener: Adapted version of the Vineland Adaptive Behaviour Scales
WEMWBS: Warwick-Edinburgh Mental Well-being Scale
Tic-P: Trimbos and iMTA questionnaire on Costs associated with Psychiatric illness

## References

[1] French L, Kennedy EMM (2018). Annual research review: Early intervention for infants and young children with, or at-risk of, autism spectrum disorder: A systematic review. J Child Psychol Psychiatry.

[2] Dawson G, Jones EJ, Merkle K, Venema K, Lowy R, Faja S, Kamara D, Murias M, Greenson J, Winter J, Smith M, Rogers SJ, Webb SJ (2012). Early behavioral intervention is associated with normalized brain activity in young children with autism. J Am Acad Child Adolesc Psychiatry.

[3] Estes A, Munson J, Rogers SJ, Greenson J, Winter J, Dawson G (2015). Long- Term outcomes of early intervention in 6-year-old children with autism spectrum disorder. J Am Acad Child Adolesc Psychiatry.

[4] Green J, Pickles A, Pasco G, Bedford R, Wan MW, Elsabbagh M, Slonims V, Gliga T, Jones E, Cheung C, Charman T, Johnson M, The British Autism Study of Infant Siblings (BASIS) (2017). Randomised trial of a parent-mediated intervention for infants at high risk for autism: longitudinal outcomes to age 3 years. J Child Psychol Psychiatr..

[5] Dawson G (2010). Randomzied controlled trial of an intervention for toddlers with autism: the Early Start Denver Model. Pediatrics.

[6] Crais ER, McComisch CS, Humphreys BP, Watson LR, Baranek GT, Reznick JS, Chirstian RB, Earls M (2014). Pediatric healthcare professionals’ views on autism spectrum disorder screening at 12–18 months. J Autism Dev Disord.

[7] Crane L, Chester JW, Goddard L, Henry LA, Hill EL (2016). Experiences of autism ddiagnosis: A survey of over 1000 parents in the United Kingdom. Autism.

[8] Snijder MIJ, Langerak IPC, Kaijadoe SPT, Buruma ME, Verschuur R, Dietz C, Buitelaar JK, Oosterling IJ (2021). Parental Experiences with Early Identification and Initial Care for their Child with Autism: Tailored Improvement Strategies. J Autism Dev Disord..

[9] Daniels AM, Mandell DS (2014). Explaining differences in age at autism spectrum disorder diagnosis: A critical review. Autism.

[10] Pijl M. S-OI.) (Vroeg)Herkenning en screening In: de Bildt A, Servatius-Oosterling I, de Jonge M (eds) Autisme bij kinderen Bohn Stafleu van Loghum, Houten. 2021.

[11] Oosterling IJ. Autism in toddlers: Aspects of early detection, diagnosis and intervention. Ipskamp Drukker The Netherlands. 2010.

[12] Mandell DS, Wiggins LD, Carpenter LA, Daniels J, DiGuiseppi C, Durkin MS, Kirby RS (2009). Racial/ethnic disparities in the identification of children with autism spectrum disorders. Am J Public Health..

[13] Rutherford M, McKenzie K, Johnson T, Catchpole C, O’Hare A, McClure I, Murray A (2016). Gender ratio in a clinical population sample, age of diagnosis and duration of assessment in children and adults with autism spectrum disorder. Autism..

[14] Boshoff K, Gibbs D, Phillips RL, Wiles L, Porter L (2018). A meta-synthesis of how parents of children with autism describe their experience of advocating for their children during the process of diagnosis. Health Soc Care.

[15] Pinto-Martin JA, Dunkle M, Earls M, Fliedner D, Landes C (2005). Developmental stages of developmental screening: steps to implementation of a successful program. Am J Public Health.

[16] Snijder MI, Kaijadoe SP, van't Hof M, Ester WA, Buitelaar JK, Oosterling IJ. Early detection of young children at risk of autism spectrum disorder at well-baby clinics in the Netherlands: Perspectives of preventive care physicians. Autism. 2021;25(7):2012–24. 10.1177/13623613211009345. 10.1177/13623613211009345 33884893

[17] Dosreis S, Weiner CL, Johnson L, Nefschaffer CJ (2006). Autism spectrum disorder screening and management practices among general pediatric providers. Dev Behav Pediatr.

[18] Pijl MK, Buitelaar JK, de Korte MW, Rommelse NN, Oosterling IJ (2018). Sustainability of an early detection program for autism spectrum disorder over the course of 8 years. Autism..

[19] Bordini D, Lowenthal R, Gadelha A, de Araujo Filho GM, Mari JJ, Paula CS (2015). Impact of training in autism for priamry care providers: a pilot study. Rev Bras Psiquiatr.

[20] Kaale A, Smith L, Sponheim E (2012). A randomized controlled trial of preschool-based joint attention intervention for children with autism. J Child Psychol Psychiatry.

[21] Kasari C, Gulsrud A, Freeman S, Paparella T, Hellemann G (2012). Longitudinal follow-up of children with autism receicing targeted interventions on joint attention and play targeted interventions on joint attention and play. J Am Acad Child Adolesc Psychiatry.

[22] Murza KA, Schwartz JB, Hahs-Vaughn DL, Nye C (2016). Joint attention interventions for children with autism spectrum disorder: a systematic review and meta-analysis. Int J Lang Commun Disord.

[23] Bruinsma Y, Minjarez M, Schreibman L, Stahmer A. Naturalistic developmental behavioral interventions for autism spectrum disorder. Baltimore: Brookes Publishing Co. 2020.

[24] Greenspan S, Wieder S. The DIR/Floortime approach to autistic spectrum disorders. In E Hollander, & E Anagnostou (Eds), Clinical manual for the treatment of autism. 2007;Arlington, VA: American Psychiatric Publishing.:179–210.

[25] Dietz C, Swinkels S, van Daalen E, van Engeland H, Buitelaar JK (2006). Screening for autistic spectrum disorder in children aged 14–15 months. II: Population screening with the Early Screening of Autistic Traits Questionnaire (ESAT). Design and general findings. J Autism Dev Disord..

[26] Van't Hof M, van Berckelaer-Onnes I, Deen M, Neukerk MC, Bannink R, Daniels AM, Hoek HW, Ester WA (2020). Novel insights into autism knowledge and stigmatizing attitudes toward mental illness in Dutch youth and family center physicians. Community Mental Health J..

[27] Laurent de Angulo MS, Brouwers-de Jong EA, Bijlsma- Schlösser JF, Pauwels JH, Steinbuch-Linstra I. Ontwikkelingsonderzoek in de jeugdgezondheidszorg het Van Wiechenonderzoek—De Baecke-Fassaert Motoriektest. Van Gorcum The Netherlands. 2008.

[28] van Berckelaer-Onnes IA, Anzion P, Sinnema H, van de Glind G. JGZ- Richtlijn Autismespectrumstoornissen: Signalering, Begeleiding en Toeleiding naar Diagnostiek. [National guideline Autismspectrumdisorder; screening, accompanying and diagnostic assessment]. Nederlands Centrum voor Jeugdgezondheidszorg. 2015.

[29] Bayley N. Bayley Scales of Infant and Toddler Development, 3rd ed: Technical Manual. San Antonio: Hartcourt Assessment; 2006.

[30] Briggs-Gowan MJ, Carter AS, Irwin JR, Wachtel K, Cicchetti DV (2004). The Brief Infant- Toddler Social and Emotional Assessment: Screening for Social-Emotional Problems and Delays in Competence. J Pediatr Psychol.

[31] Bralten JVH KJ, Martens MB, Galesloot TE, Kiemeny LA, Buitelaar JK, Muntjewerff JW, Franke B, Poelmans G, Arias-Vasquez A (2018). Autism spectrum disorders and autistic traits share genetics and biology. Molecular Psychiatry..

[32] Adamson LBBR, Suma K (2020). The joint engagement rating inventory (JERI): Adaptation for Karakter Technical Report 25i..

[33] Grzadzinski RCT, Colombi C, et al. Measuring changes in social communication behaviors: preliminary development of the Brief Observation of Social Communication Change (BOSCC). J Autism Dev Disord. 2016;46(2464–2479). 10.1007/s10803-016-2782-9 27062034

[34] Lord CRM, DiLavore P (2012). Autism Diagnostic Observation Schedule: Second Edition (ADOS-2).

[35] Fenson L, Dale P, Reznick JS, Thal D, Bates E, Hartung JP, Pethick S, Reilly JS (1993). The MacArthur Communicative Development Inventories: User’s guide and technical manual.

[36] Fenson L, Pethick S, Renda C, Cox JL (2000). Short-form versions of the MacArthur Communicative Developmental Inventories. Appl Psycholinguist.

[37] Sparrow SS, Cichetti DV, Balla DA (2005). Vineland adaptive behavior scales.

[38] van Duijn G, Dijkxhoorn Y, Noens I, Scholte E, van Berckelaer-Onnes I (2009). Vineland Screener 0–12 years research version (NL). Constructing a screening instrument to assess adaptive behaviour. Int J Methods Psychiatr Res.

[39] Tennant R, Hiller L, Fishwick R, Platt S, Weich S, Parkinson J, Secker J, Stewart- Brown SL. The Warwick-Edinburgh Mental Well-being Scale (WEMWBS) : development and UK validation. Health and Quality of Life Outcomes. 2007;5(63). 10.1186/1477-7525-5-63 PMC2222612 18042300

[40] Vermulst A, Kroes G, De Meyer R, Nguyen L, Veerman JW. Opvoedingsbelastignsvragenlijst (OBVL) [ Parenting stress questionnaire]. Nijmegen: Praktikon. 2012.

[41] Killmeyer SKL (2017). Parent training and joint engagement in young children with autism spectrum disorder. Autism Dev Language Impairments..

[42] De Meyer RE, Janssen J, Veerman JW. Handleiding Beoordelingsschaal Tevredenheid en Effect (BESTE). Nijmegen: Praktikon; 2004.

[43] Tan SS, Bouwmans CAM, Rutten FF, Hakkaart-van Roijen L (2012). Update of the Dutch Manual for Costing in Economic Evaluations. Int J Tech Assess Health Care..

[44] Kasari C, Siller M, Huynh LN, Shih W, Swanson M, Hellemann GS, Sugar CA (2014). Randomized controlled trial of parental responsiveness intervention for toddlers at high risk for autism. Infant Behav Dev.

