# Supplementary material for: Social COmmunication Program supported by E-health (SCOPE) for infants and toddlers at elevated likelihood of autism spectrum disorder: study design of a cluster randomized controlled trial
Source: BMC Psychiatry. 2022 Dec 8;22:772. doi: 10.1186/s12888-022-04351-x (PMC9733381; doi:10.1186/s12888-022-04351-x)
Supplement: Supplementary file 1 — Additional file 1: Appendix A. Informed Consent. [file 12888_2022_4351_MOESM1_ESM.pdf]

Additional file 1: Parental satisfaction questionnaire

1. Who filles in this questionnaire?

- ❑ Biological mother
- ❑ Biological father
- ❑ Stepmother
- ❑ Stepfather
- ❑ Adoptive mother
- ❑ Adoptive father
- ❑ Foster mother
- ❑ Foster father
- ❑ Other, namely____

2. What were initial concerns regarding your child's development?
Multiple options are possible.

- ❑ Language development (e.g. no babbling, no use of short sentences, previously spoken words that disappeared, etc.)
- ❑ Motor development (e.g. late sitting, walking and/or crawling, clumsy movement, difficulty with fine motor skills such as holding a pencil, etc.)
- ❑ Social interaction (e.g. difficulty having fun together, not smiling at others, not waving, eye contact etc.)
- ❑ Behavioral concerns (e.g. anger attacks and/or tantrums, anxious behaviour, self-determining behaviour, etc.)
- ❑ Medical concern (e.g. epilepsy, metabolic disease, etc.)
- ❑ Potty training concerns
- ❑ Eating problems (e.g. hypersensitivity to texture, smell and/or taste of the food, wanting to eat only certain foods, not daring to taste new things, etc.)
- ❑ Sleeping problems
- ❑ Other, namely____

3. With which care provider have you discussed these concerns?

- ❑ General practitioner
- ❑ Preventive care physician /nurse at the well-baby clinic
- ❑ Other, namely ____

A number of statements now follow. To what extent do the following statements apply to your situation?

4.1. I felt heard and understood by the counselor (as referred to in question 3) with whom I discussed my initial concerns.

Not at all Very N/A

1 2 3 4 5 0

Space for any comments:

____

____

____

4.2 The care provider (as referred to in question 3) with whom I discussed my initial concerns acted properly and in accordance with my wishes/needs (e.g. immediately referring for diagnostics because I wanted to/waiting for a referral because this suited my wish).

Not at all Very N/A

1 2 3 4 5 0

Space for any comments:

____

____

____

5. Has your child been referred for help (in any form)?

- ❑ No → Proceed to question 7.
- ❑ Yes → Proceed to question 6.

6. Which care provider did you turn to for help? Multiple answers are possible

- ❑ Social teams
- ❑ Integrated early care
- ❑ First-line psychologist practice
- ❑ Specialized mental health care institution
- ❑ Pediatricia
- ❑ ENT specialist
- ❑ Neurologist
- ❑ Speech therapist
- ❑ Physiotherapist
- ❑ Occupational therapist
- ❑ BEAR professional
- ❑ Specialized childcare
- ❑ Other, namely____

7. Has your child been diagnosed?

- ❑ Yes → proceed to question 8
- ❑ Diagnostic testing is currently underway → Proceed to question 8
- ❑ My child is on the waiting list for diagnostic testing → Proceed to question 1
- ❑ No, and no research has been carried out → Proceed to question 11
- ❑ No, no diagnosis has been made after examination → Proceed to question 11

8. What diagnosis has your child received? If diagnostic testing is currently still ongoing: which diagnosis is being considered?

For example, an Autism Spectrum Disorder, ADHD, ADD, etc.

____

9. How old was your child when he/she was diagnosed? If diagnostic testing is currently underway, we would like to ask you to enter the age of your child at the start of the diagnostic investigation. Try to be as accurate as possible.

___________ years and ___________ months old.

10. Before a child receives a diagnosis or diagnostic testing takes place, there is often an entire process in which various care providers are visited.

How many different care providers did you and your child see/visit before your child received a diagnosis or a diagnostic investigation was started? By different care providers we mean a speech therapist, psychologist, physiotherapist, etc. The general practitioner and youth doctor are not included here.

How many different care providers have you and your child visited? And which care providers were these?

- ❑ 0
- ❑ 1, namely ________________________
- ❑ 2, namely ________________________
- ❑ 3, namely ________________________
- ❑ 4, namely ________________________
- ❑ 5, namely ________________________
- ❑ More than 5, namely________________________

11. Has your child received training, therapy or treatment, or is your child receiving training, therapy or treatment?

- ❑ Yes → Proceed to question 12.
- ❑ No Proceed to question 15.

12. Which training, therapy or treatment has your child received or will your child receive? Multiple options are possible.

- ❑ Social skills training
- ❑ PRT (pivotal response treatment)
- ❑ Floorplay
- ❑ DTT (discrete trial teaching) / ABA (applied behavior analysis)
- ❑ JASPER Training
- ❑ Other, namely ____
- ❑ Not applicable

13. How old was your child when training, therapy or treatment was first started? Try to be as accurate as possible.

___________ years and ___________ months old.

14. Looking back, how satisfied are you with the above treatment(s) your child received?

Treatment 1:____

Not at all Very

1 2 3 4 5

Space for any comments:

____
____
____

Treatment 2: ____

Not at all Very

1 2 3 4 5

Space for any comments:

____
____
____

Treatment 3: ____

Not at all Very

1 2 3 4 5

Space for any comments:

____
____
____

Treatment 4: ____

Not at all Very

1 2 3 4 5

Space for any comments:

____
____
____

15. Have you had treatment or counseling contacts related to the upbringing and support of your child?

- ❑ Yes → Proceed to question 16.
- ❑ No → Proceed to question 18.

16. What therapy or guidance contacts related to the upbringing and support of your child have you received?

- ❑ Participation in parent group, in which explanation about ASD was given
- ❑ Parent guidance
- ❑ Home support
- ❑ Video home training
- ❑ Family therapy

- ❑ Other, namely ____

17. Looking back, how satisfied are you with the above therapy/guidance contacts?

Therapy 1:____________________________________

Not at all Very

1 2 3 4 5

Space for any comments:

____
____
____

Therapy 2:____________________________________

Not at all Very

1 2 3 4 5

Space for any comments:

____
____
____

Therapy 3:____________________________________

Not at all Very

1 2 3 4 5

Space for any comments:

____
____
____

18. To what extent has the overall care you or your child received met your wishes?

Not at all Very

1 2 3 4 5

19. Looking back on the entire process (from initial concerns to now), what are you most satisfied with?

20. Looking back at the entire process (from initial concerns to now), what would you like to see improved?
